# Global dust Detection Index (GDDI); a new remotely sensed methodology for dust storms detection

**DOI:** 10.1186/2052-336X-12-20

**Published:** 2014-01-09

**Authors:** Mehdi Samadi, Ali Darvishi Boloorani, Seyed Kazem Alavipanah, Hossein Mohamadi, Mohamad Saeed Najafi

**Affiliations:** 1 Department of Remote Sensing and GIS, Faculty of Geography, University of Tehran, Tehran, Iran; 2 Geoinformatics Research Institute (GRI), University of Tehran, Tehran, Iran; 3 Department of Physical Geography, Faculty of Geography, University of Tehran, Tehran, Iran; 4 Faculty of Geography, University of Tabriz, Tabriz, Iran

**Keywords:** Remote sensing, Dust detection index, MODIS

## Abstract

Dust storm occurs frequently in arid and semi-arid areas of the world. This natural phenomenon, which is the result of stormy winds, raises a lot of dust from desert surfaces and decreases visibility to less than 1 km. In recent years the temporal frequency of occurrences and their spatial extents has been dramatically increased. West of Iran, especially in spring and summer, suffers from significant increases of these events which cause several social and economic problems. Detecting and recognizing the extent of dust storms is very important issue in designing warning systems, management and decreasing the risk of this phenomenon. As the process of monitoring and prediction are related to detection of this phenomenon and it's separation from other atmospheric phenomena such as cloud, so the main aim of this research is establishing an automated process for detection of dust masses. In this study 20 events of dust happened in western part of Iran during 2000–2011 have been recognized and studied. To the aim of detecting dust events we used satellite images of MODIS sensor. Finally a model based on reflectance and thermal infrared bands has been developed. The efficiency of this method has been checked using dust events. Results show that the model has a good performance in all cases. It also has the ability and robustness to be used in any dust storm forecasting and warning system.

## Introduction

Every year in Iran, several natural hazards occur which cause social, economic and environmental damages. Western dust storms, i.e. the dust coming from western neighbors of Iran, are one of these hazards which have been increased in both spatial and temporal aspects during last decade.

Dust storms are, in most cases, the result of turbulent winds which raise large quantities of dust from land surfaces and reduce visibility to less than 1 km [1]. They reach concentrations in excess of 6000 μg/m^3^ in severe events [2]. Dust storms are generated from regions that are mainly deserts, dry lakebeds and semi-arid desert regions [3]. They can carry large quantity of dust and move forward to destroy crop plants, ruin the mining and communication facilities, reduce visibility and disturb human’s daily activities. They also impact the air and ground transportation. They pollute the atmosphere and reduce air quality, influence cloud formation [4], obscure the sunlight, and reduce the temperature [5]. They also can accelerate the desertification procedure [6]. Their direct effects on human health are mainly depicted in breathing difficulties [7].

Over the past decades, Middle East dust storms have caused many problems for the residents of South and Southwest regions of Iran. During the recent years, there has been an increase in the trend of dust storm activities in this region, especially in spring and summer [8]. Now, this trend is changing into the main persistent environmental problem in Iran and the Middle East region. Middle East dust storms have great impacts on the quality of the inhabitant’s lives, visibility and transportation, microclimate, ecosystem, communication systems, and consequent crisis, such as eco-social and environmental problems in the west and southwest of Iran [9].

Detecting dust phenomena, identifying their sources and surveying about their movements and situation can help planners and decision makers in planning and controlling to reduce damages of this phenomena. Traditional ground measurement cannot monitor and forecast dust storm efficiently, because of low temporal and spatial resolutions [10], therefore, it can’t be enough for such studies. While satellite remote sensing can be more effective because of suitable spatial and temporal resolutions and providing observations of dust aerosols from regional to global scales [11]. Remote sensing allows for better tracking of regional and global distribution of aerosols, which are extremely dynamic in nature [12]. By using remote sensing, detecting and mapping of dust events, dust transport pathways, identifying dust source regions [13] and forecasting the next destination of them [10] are more faster, easier and economical.

Several studies have been done about identifying dust source regions using satellite imagery [13-16]. Also in case of using remote sensing and satellite imagery for detecting dust storms several methods have been developed since 1970. Some of them use visible and infrared spectrum [17], some use thermal infrared [18-21], while some techniques use composite of reflective and thermal spectrum [22,23] and some use a composite of thermal and microwave spectrum[24] to detect dust and separating it from other atmospheric phenomena. Ackerman (1989) used brightness temperature difference (BTD) 3.7 and 11 μm spectrum to detect and monitor dust storms. He developed a tri-spectral (8, 11 and 12 μm) technique later for detecting dust over water and for distinguishing dust plumes from water/ice clouds [18]. The negative differences of BTD (11–12 μm) are useful for dust storms detection and sources identification.

Qu et al. (2006) [17] used Normalized Dust Difference Index (NDDI) to monitor Asian dusts. The NDDI can be written as equation (1).

(1) $$\mathrm{NDDI}=\left(\rho 2.13\;\mu m-\rho 0.469\;\mu m\right)/\left(\rho 2.13\;\mu m+\rho 0.469\;\mu m\right)$$

Where, *ρ*2*.*13-*μ*m and *ρ*0*.*469-*μ*m are reflectance at the top of atmosphere in the 2.13 and 0.469 *μ*m bands, respectively.

Hao and Qu (2007) [20] used thermal-infrared dust index (TDI) for 20, 30, 31 and 32 bands of MODIS for detecting and monitoring dust storms. The proposed approach used thermal bands only, so it has the capability to detect dust at night time. TDI is mathematically defined in equation (2).

(2) $$\begin{array}{ll} \mathrm{TDI}= & c0+c1*\mathrm{BT}20+c2*\mathrm{BT}30+c3\\ & *\mathrm{BT}31+c4*\mathrm{BT}32 \end{array}$$

Where, BT20, BT30, BT31 and BT32 are brightness temperature of 20, 30, 31 and 32 bands of MODIS data, respectively. c0, c1, c2, c3 and c4 are coefficients. (Table 1) lists the values of these coefficients.

**Table 1:** Coefficients of equation (2)

**C4**	**C3**	**C2**	**C1**	**C0**	**Coefficient**
0.5883	-0.7068	0.0260	0.1227	-7.9370	**Value**

Some other studies are carried out by MODIS images [10-12,25-27], TOMS and OMI (Ozone Monitoring Instrument [28], AVHRR images (Advanced Very High Resolution Radiometer) [29,30], METEOSAT data [31], and SeaWIFS images (Sea-viewing Wide Field-of-View Sensor) [32] for dust storms detection, discrimination and monitoring purposes with some successes.

By considering almost all developed methodologies, there are common limitations in them. First, while they have good abilities for dust detection over lands, they cannot do the same over water bodies. Second, they have problems with seasonal changes and they need different thresholds. Third, they almost have problems with dust discrimination from other objects like clouds, water and land soil surface. Therefore, the main objective of this research is the development of a global methodology which resolves mentioned problems. This methodology is able to detect dust storms in all seasons with no need to threshold and discriminates dusts from other objects. The developed methodology we called “Global Dust Detection Index (GDDI)” resolves all of these problems in previously developed methodologies.

## Materials and methods

### Study area

**Figure 1:**
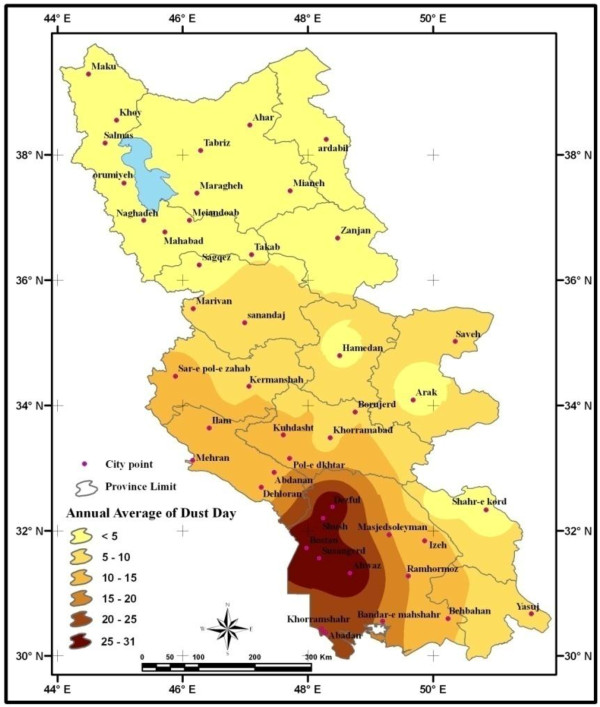
Study area and annual distribution of dust during 2000–2011.

Study area of this research is western part of Iran which is close to internal and transboundary sources of dust which are exposed to several dust systems and many dust storms occur in these areas every year. Surveying meteorological data from 2000–2011 indicates that in some cities like Ahwaz, Dezful, Susangerd, Bostan and Shush the annual average of days with dust is more than 31 days. In these days the visibility was less than 1000 m. Figure 1 shows the average of days with annual dust events which happened in western part of Iran.

### Meteorological data

Almost all western and south western parts of Iran which are affected by local, regional and global dust storms are considered in this study. The synoptic data from 27 stations are provided by the Islamic Republic of Iran Meteorological Organization (IRIMO). The dusts are identified based on two criteria: first, to be measured in three stations simultaneously and second visibilities less than 1000 meter is reported. In this way 20 cases of dust events (Table 2) were selected.

**Table 2:** Dust event days from 2000-2011

**Date (yy/mm/dd)**	**Event**	**Date (yy/mm/dd)**	**Event**
2008/07/01,02	11	2000/06/11	1
2008/09/16,17	12	2003/03/26,27	2
2008/10/01	13	2004/05/14,15	3
2009/06/05	14	2005/07/04,05	4
2009/07/04,05	15	2005/08/08,09	5
2009/07/30	16	2007/03/03	6
2010/03/04	17	2007/05/17	7
2011/03/04	18	2007/07/09	8
2011/04/12,13	19	2007/07/18	9
2011/06/02	20	2008/05/25,26	10

### Remote sensing images

Satellite remote sensing is advantageous in monitoring the significant spatial-temporal variations of dust storms [33,34]. Dust phenomena can be detected by remote sensing in different spectral channels. Although the accuracy of results depends on various parameters such as the spatial, spectral and radiometric resolutions of satellite images, the methodology used spectral bands, defined thresholds, weather and atmospheric conditions, clouds and etc. Data from the Moderate Resolution Imaging Spectroradiometer (MODIS) were used in this study. MODIS makes observations using 36 spectral bands with wavelengths from 0.41 to 14.4 μm and nadir spatial resolutions of 0.25, 0.5, and 1 km [13].

MODIS is currently operating onboard the NASA Earth Observing System (EOS) Terra and Aqua satellites, launched in December 1999 and May 2002, respectively [13]. MODIS images from both Terra and Aqua satellites were obtained in Level 1 from Atmosphere Archive and Distribution System (LAADS; http://ladsweb.nascom.nasa.gov/).

In order to accurately decide the bands and thresholds in the algorithm of dust detection, more than 20 dust storm events occurred in the west part of Iran during 2000–2011 were collected as training data for spectral analysis. Due to limitations of pages and paper only 4 dust storm events are selected as example cases.

### Methods

**Figure 2:**
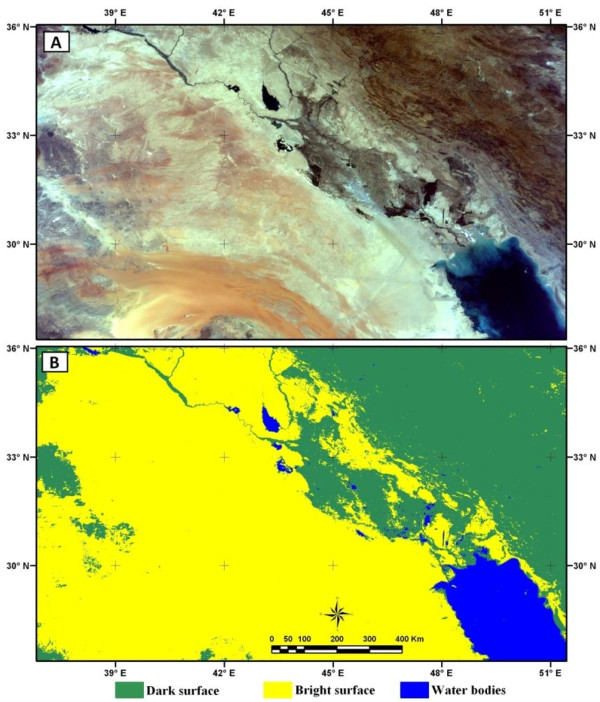
**Surface types of study area. (A) MODIS true color image on 25 July 2007 and (B) land cover extracted from MODIS image in 3 classes**.

As the behavior of dust storms is not the same over different land covers and water bodies, first the land cover map of the study area must be produced. Using MODIS images, in clear days, three main land cover classes were separated: bright land cover, dark land cover and water bodies. The bright surfaces like deserts and plains have much higher radiances than dark surfaces like mountains and vegetation in satellite images. In addition to the map of water bodies, based on an equation (Equ. 3) from Rimer et al., (2005) [35] these two land cover classes were separated. Figure 2 shows MODIS true color image of study area and land cover in 3 classes extracted from MODIS image. In this figure dark green color shows dark surfaces, yellow color indicates bright surfaces and blue color shows water bodies.

(3) $$0.01\leq {\mathrm{Ref}}_{\;2.13}\leq .25$$

Where, Ref_2.13_ is the reflectance of 2.13 μm of MODIS data. If the equation be true, the surface is dark.

To achieve and modeling the spectral behavior of different objects and also discriminating them from each other, the training pixels were collected. In this procedure the dusty pixels over different land covers i.e. bright and dark land covers and water bodies were collected. This procedure was carried out for all images, separately and almost all MODIS bands were used. Finally the useful bands based on our and other studies’ results were selected. For each class in the scene, about thirty thousand training pixels were collected. Then, the statistical mean and standard deviation of samples for seven classes, i.e. clouds, bright surfaces, dust over bright surfaces, dark surface, dust over dark surfaces, water and dust over water bodies, were calculated and spectral curve of these classes were drawn.

### Spectral curves and indices

**Figure 3:**
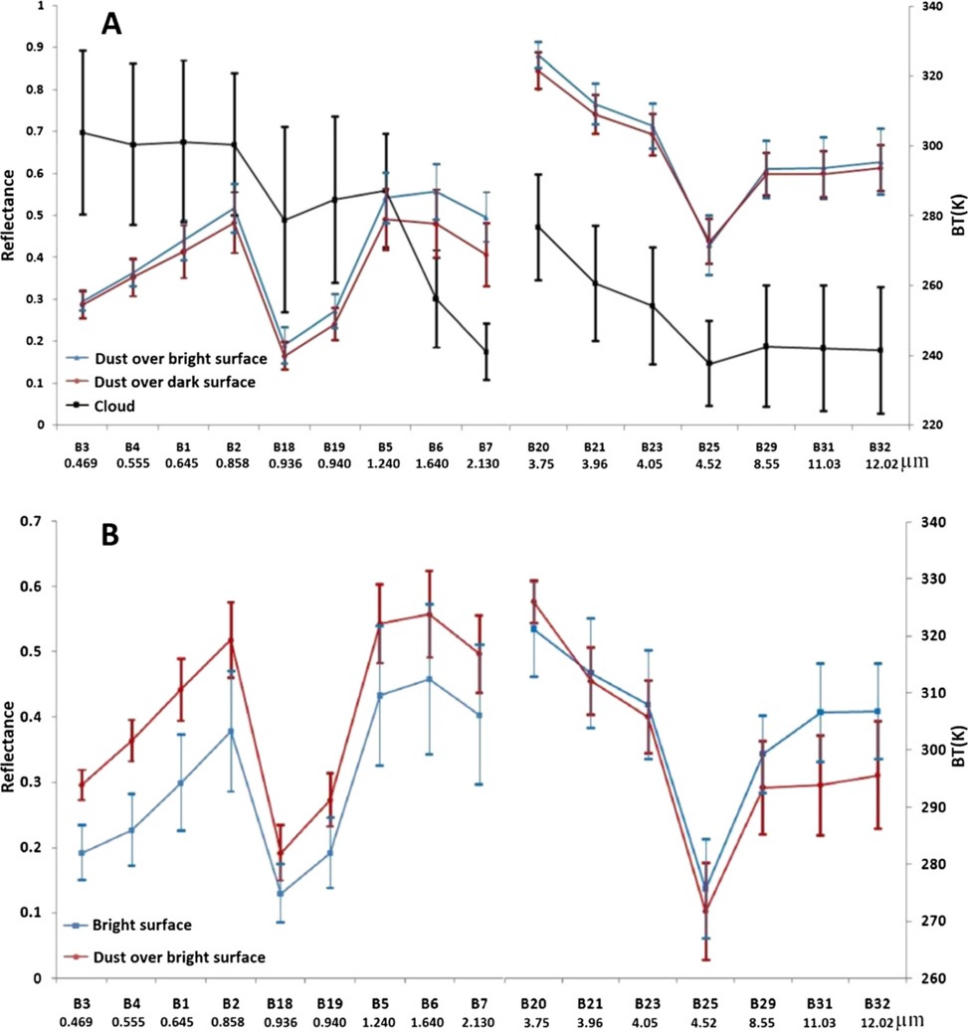
**(A) Spectral curves of dust over bright surface, dust over dark surface and cloud in optical and thermal bands, (B) Spectral curves of bright surface and dust over dark surface in optical and thermal bands**.

To draw spectral curve of defined classes, training samples were taken from selected bands. After collecting training pixels, statistical means and standard deviations were calculated for each class in all individual bands, and spectral curves were drawn. Investigating spectral behavior curves of classes shows that clouds have high reflectance in band 3, and low reflectance in band 7 of MODIS; while dust has reverse mode with high reflectance in band 7 and low in band 3. In thermal spectrum, clouds have much lower brightness temperature than dust (Figure 3-A). Therefore, these differences in behaviors of clouds and dust are useful for distinguishing them from each other.

Qu et al (2006) [17] developed the NDDI index to detect dust (Equ. 4).

(4) $$\mathrm{NDDI}=\left(B7-B3\right)/\left(B7+B3\right)$$

Where, B3 is reflectance in band 3 and B7 is reflectance in band 7. Because of noticeable difference in brightness temperature between clouds and dust in thermal spectrum, Ackerman (1997) [18] proposed a method to differentiate dust from clouds which used brightness temperature difference of 11 and 12 μm (band 31 and 32 of MODIS image, respectively). So brightness temperature difference in 11 and 12 μm (BT31-BT32) and the NDDI index can separate dust from clouds.

To separate bright surface from dust over bright surface, BTD in 3.7 and 11 (band 20 and 31 respectively) μm can be used. These two bands behave inversely for dust over bright surface in compare to bright surface. Dust over bright surface has higher BT than bright surface in band 20, while bright surface has higher BT than dust over bright surface in band 31 (Figure 3-A). After considering all datasets more precisely, we could find a relationship between bands 4 and 7 for discrimination of dusty from none-dusty pixels over bright surface (Figure 3-B). So to separate these two phenomena, we used the following index:

(5) $$\left(B7-B4\right)/\left(B7+B4\right)$$

Where, B4 and B7 are the reflectance of bands 4 and 7 in MODIS L1B data, respectively. So using equation 5 and brightness temperature difference at 3.7 and 11 μm (BT20 – BT31) we can separate dust from bright surface.

**Figure 4:**
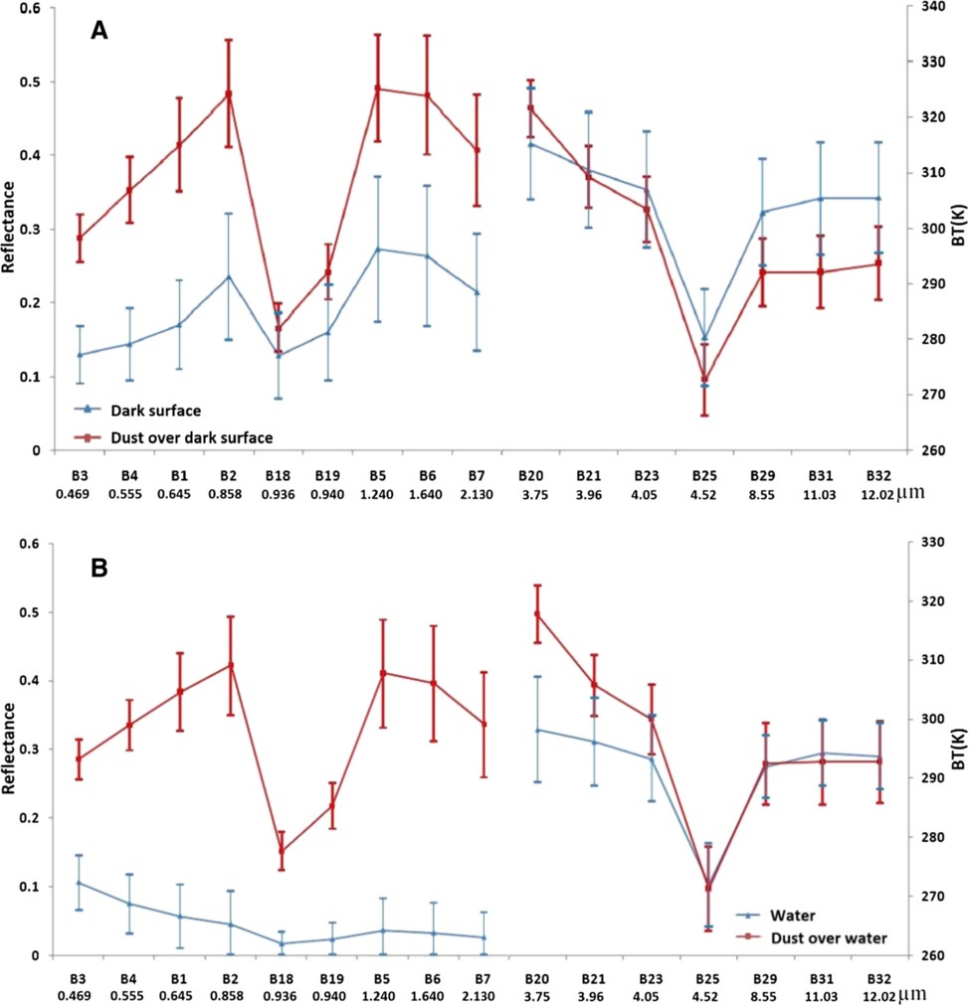
**Spectral curves of some studied phenomena in reflected and thermal bands. (A) dark surface, dust over dark surface and (B) water and dust over water**.

Dark surfaces have lower BT than dust over dark surface in band 20, while in band 31 dark surfaces have higher BT than dust over dark surface (Figure 4-A). Therefore, BTD at band 20 and 31 can separate dust from dark surfaces.

In the reflectance spectrum these two phenomena show more separation and this difference was maximized in band 2 and minimized in band 18 (Figure 4-A). So the difference of these two bands can separate the two phenomena. The spectral properties of water show a low reflectance for none-dusty pixels over water bodies and this property for dusty pixels is high in almost all bands. Band 2 shows the highest separation for the reflectance of none-dusty from dusty pixels over water bodies. Comparatively, this separation in band 1 is lower (Figure 4-B). The location of bands 1 and 2 of MODIS in the red and NIR portions of spectrum let us to adapt the NDVI = (B2-B1)/(B2 + B1) for bounding the pixels in none-dusty from dusty ones over water bodies.

**Figure 5:**
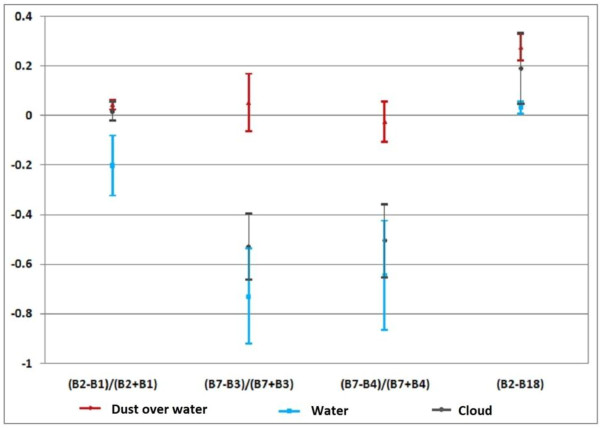
Statistical analysis of dust over water, water and cloud in 4 defined indices for deciding thresholds.

Finally to define the threshold of the above mentioned indices, sample pixels using the defined indices on MODIS L1B data were collected and statistical mean and standard deviation of training pixels were calculated. We collect training pixels from all images with almost the same number of pixels for all classes (water, cloud, bright surface, dark surface, dust over bright surface, dust over dark surface and dust over water bodies). As shown in Figure 5, the amount of NDVI for water is less than zero most of the time. Therefore, a threshold equal to zero will separate water from other objects. The amount of NDDI for clouds is also less than zero. Thus, zero threshold for this index is a good boundary for separating clouds from others.

**Figure 6:**
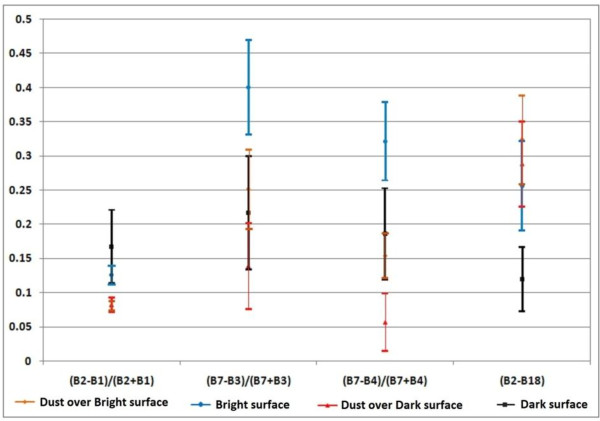
Statistical analysis of dust over bright surface, bright surface, dust over dark surface and dark surface in 4 defined indices for deciding thresholds.

Experiments show that the bright surfaces have amounts higher than 0.25 in the adapted (B7-B4)/(B7 + B4) index. In the defined B2-B18 index, the dark surfaces have a threshold less than 0.2. These two thresholds were adapted for separate bright and dark surfaces from other classes, respectively (Figure 6).

**Figure 7:**
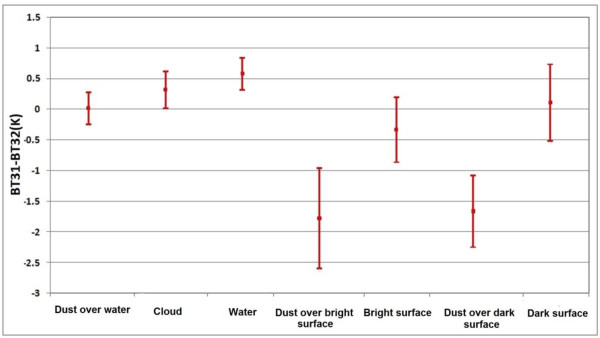
Statistical properties of dust over water, cloud, water, dust over bright surface, bright surface, dust over dark surface and dark surface in BT31-BT32 to decide about thresholds.

**Figure 8:**
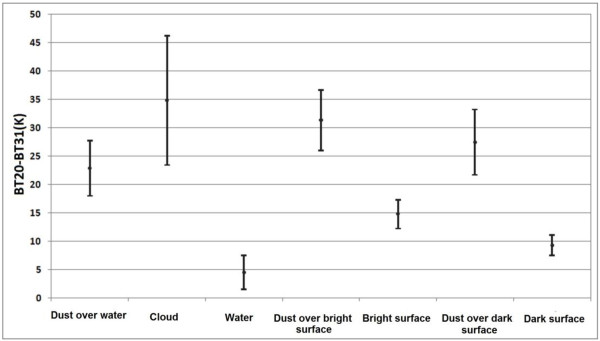
Statistical properties of samples of dust over water, cloud, water, dust over bright surface, bright surface, dust over dark surface and dark surface in BT20-BT31 to decide about thresholds.

The results of BTD (31–32) show that the best threshold to separate clouds is the amounts higher than zero (Figure 7). Results also show that for the BTD (20–31) the amounts higher than 20 and 15 Kelvin, are dust over bright and dark surfaces, respectively (Figure 8).

Due to the different nature of dust detection over water bodies, the amount of threshold for some indices like NDDI could be changeable. The existence of icy clouds is also a problem. Experimentally a threshold more than one in the BTD (31–32) was adapted for icy clouds separation from the dust in the image.

**Figure 9:**
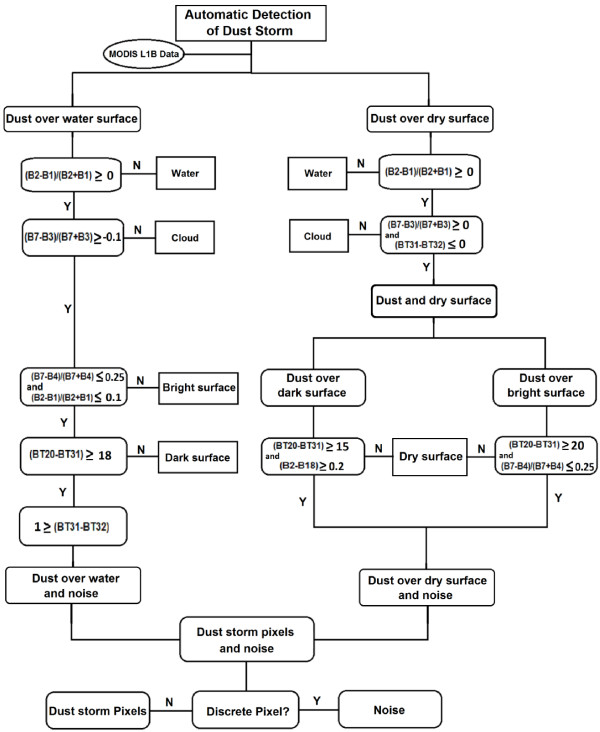
Flowchart of Global Dust Detection Index (GDDI).

As shown in the flowchart (Figure 9), MODIS L1B data defined as model input. The whole procedure is divided into two parts: dust over land and dust over water. In dust detection over land, after removing water and clouds, the land is divided into dark and bright surfaces. By using the defined indices, these two parts separate from image and what remains is dust and noise. In the same procedure dusts over water are also discriminated. So, all features separate from image step by step. At the end, the results of both will be combined for making a dust storm map. We also face some single pixels that are noise. Using median filter the noises were removed from the final map.

## Results and discussion

In order to evaluate the developed methodology 20 dust events were examined and four of them which happened in the west part of Iran are presented here (Table 3).

**Table 3:** Dust event cases to evaluate the developed dust detection algorithm

**Date**	**Used satellite image**
May 17, 2007	Terra-MODIS
July 1, 2008	Terra-MODIS
July 5, 2009	Aqua-MODIS
April 13, 2011	Terra-MODIS

**Figure 10:**
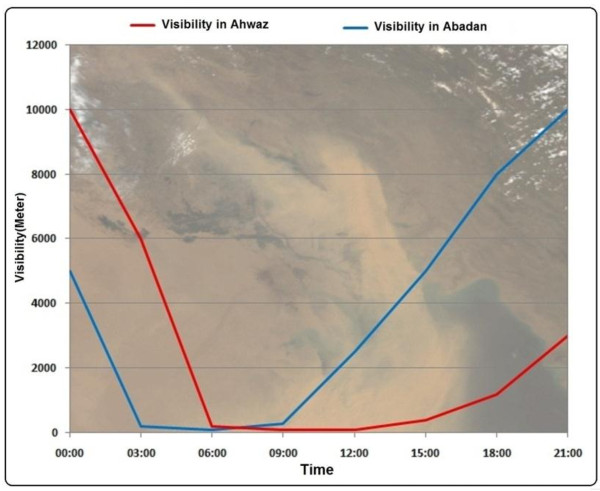
Visibility curves for Ahwaz and Abadan by meteorology stations on 17 May 2007.

**Figure 11:**
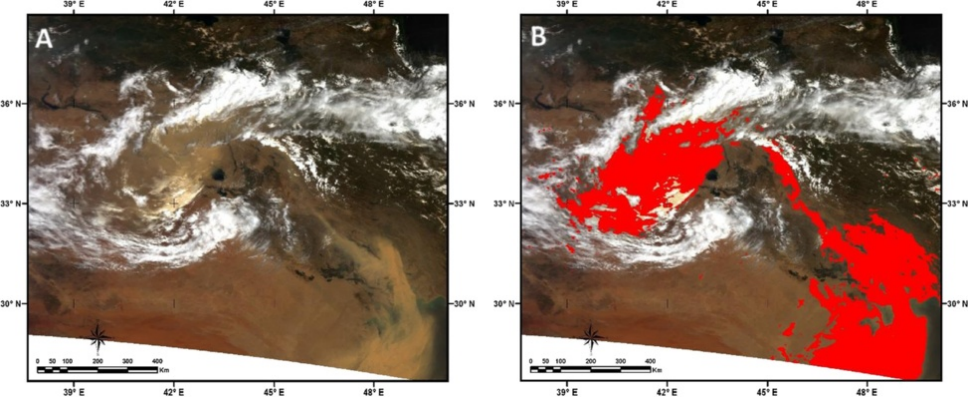
**Evaluation of the developed dust detection method. (A) MODIS true color image on 17 May 2007 and (B) Detected dust event using developed method**.

**Figure 12:**
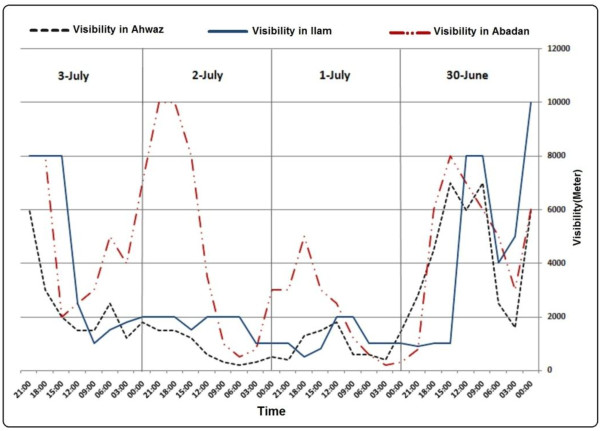
Visibility curves for Ahwaz, Ilam and Abadan on 30 June, 1, 2 and 3 July 2008.

**Figure 13:**
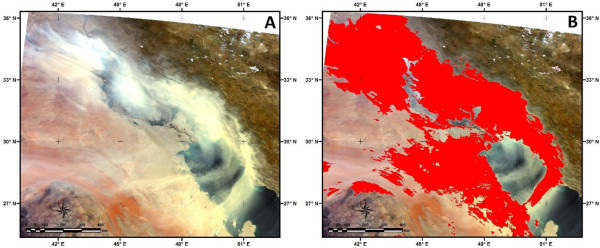
**Evaluation of the developed dust detection method. (A)** MODIS true color image on 1 July 2008 and **(B)** Detected dust event using developed method.

**Figure 14:**
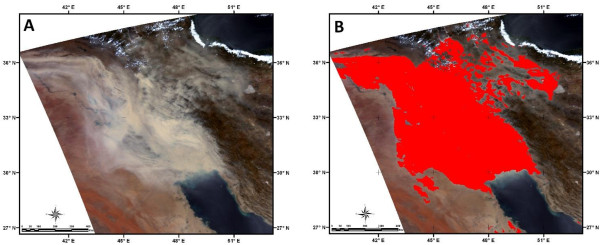
**Evaluation of the developed dust detection method. (A)** MODIS true color image on 5 July 2009 and **(B)** Detected dust event using developed method.

**Figure 15:**
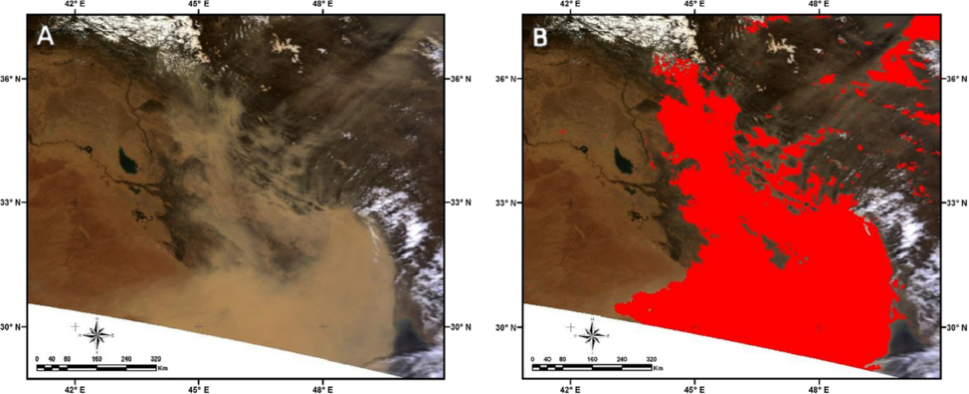
**Evaluation of the developed dust detection method. (A)** MODIS true color image on 13 April 2011 and **(B)** Detected dust event using developed method.

During these events some cities of western parts of the country were affected. In dust event of May 17, 2007 the reported visibility in cities like Ahwaz and Abadan was about 100 meters (Figure 10). In other cases dust events affected some provinces of Iran and caused some problems in human health, construction and transportation. Using GDDI we displayed the affected area in satellite image. Figure 11 shows MODIS true color image on 17 May 2007 and affected areas detected by developed method**.** Figure 12 shows Visibility curves for Ahwaz, Ilam and Abadan on 30 June, 1, 2 and 3 July 2008. This figure shows visibility reduced to less than 200 meters in some days. Figures 13, 14 and 15 show MODIS true color image and detected dust event using developed method on 1 July 2008, 5 July 2009 and 13 April 2011 respectively. In cases of 2007, 2008 and 2011 Terra-MODIS and in the case of 2009 Aqua-MODIS image were used to evaluate the developed dust detection algorithm.

## Conclusion

In this work the Global Dust Detection Index (GDDI) was developed for automatic dust storm detection using satellite images. Its abilities were evaluated by MODIS L1B data. It enjoys the optical and thermal portions of the electromagnetic spectrum. Apart from some experimental indices, we explored the BTD and NDDI. Compared to previous methodologies for detection of dust, the GDDI has no need of threshold. Being able to work in all climatic conditions is another characteristic of GDDI which makes it preferable. It also is able to simultaneously detect dusts over land surfaces and water bodies.

## Competing interests

The authors declare that they have no competing interests.

## Authors’ contributions

The work presented here was carried out in collaboration between all authors. All authors read and approved the final manuscript.
